# Peginterferon beta-1a improves MRI measures and increases the proportion of patients with no evidence of disease activity in relapsing-remitting multiple sclerosis: 2-year results from the ADVANCE randomized controlled trial

**DOI:** 10.1186/s12883-017-0799-0

**Published:** 2017-02-10

**Authors:** Douglas L. Arnold, Peter A. Calabresi, Bernd C. Kieseier, Shifang Liu, Xiaojun You, Damian Fiore, Serena Hung

**Affiliations:** 1 Montreal Neurological Institute, McGill University, Montreal, QC Canada; 2 NeuroRx Research, Montreal, QC Canada; 3 Department of Neurology, Johns Hopkins University, Baltimore, MD USA; 4 Department of Neurology, Medical Faculty, Heinrich-Heine University, Düsseldorf, Germany; 5 Biogen, 225 Binney St, Cambridge, MA USA

**Keywords:** Clinical trial, Phase 3, Multiple sclerosis, Relapse-remitting multiple sclerosis, Peginterferon beta-1a, Pegylation, Interferon, Magnetic resonance imaging, NEDA, No evidence of disease activity

## Abstract

**Background:**

Subcutaneous peginterferon beta-1a has previously been shown to reduce the number of T2-hyperintense and gadolinium-enhancing (Gd+) lesions over 2 years in patients with relapsing-remitting multiple sclerosis (RRMS), and to reduce T1-hypointense lesion formation and the proportion of patients showing evidence of disease activity, based on both clinical and radiological measures, compared with placebo over 1 year of treatment. The objectives of the current analyses were to evaluate T1 lesions and other magnetic resonance imaging (MRI) measures, including whole brain volume and magnetization transfer ratio (MTR) of normal appearing brain tissue (NABT), and the proportions of patients with no evidence of disease activity (NEDA), over 2 years.

**Methods:**

Patients enrolled in the ADVANCE study received continuous peginterferon beta-1a every 2 or 4 weeks for 2 years, or delayed treatment (placebo in Year 1; peginterferon beta-1a every 2 or 4 weeks in Year 2). MRI scans were performed at baseline and Weeks 24, 48, and 96. Proportions of patients with NEDA were calculated based on radiological criteria (absence of Gd + and new/newly-enlarging T2 lesions) and clinical criteria (no relapse or confirmed disability progression) separately and overall.

**Results:**

Peginterferon beta-1a every 2 weeks significantly reduced the number and volume of T1-hypointense lesions compared with delayed treatment over 2 years. Changes in whole brain volume and MTR of NABT were suggestive of pseudoatrophy during the first 6 months of peginterferon beta-1a treatment, which subsequently began to resolve. Significantly more patients in the peginterferon beta-1a every 2 weeks group compared with the delayed treatment group met MRI-NEDA criteria (41% vs 21%; odds ratio [OR] 2.56; p < 0.0001), clinical-NEDA criteria (71% vs 57%; OR 1.90; p < 0.0001) and achieved overall-NEDA (37% vs 16%; OR 3.09; p < 0.0001).

**Conclusion:**

Peginterferon beta-1a provides significant improvements in MRI measures and offers patients a good chance of remaining free from evidence of MRI, clinical and overall disease activity over a sustained 2-year period.

**Trial registration:**

ClinicalTrials.gov: NCT00906399; Registered on: May 20, 2009.

**Electronic supplementary material:**

The online version of this article (doi:10.1186/s12883-017-0799-0) contains supplementary material, which is available to authorized users.

## Background

Interferon beta-1a is a therapeutic protein that has been used for many years as an effective treatment for multiple sclerosis (MS). A limitation is the need for frequent administration and associated injection-site adverse events [1]. Attachment of polyethylene glycol (PEG) molecules (pegylation) has been shown to improve the pharmacokinetic and pharmacodynamic properties of protein therapeutics, including interferon beta-1a [2, 3]. The approved dosing schedule of pegylated interferon beta-1a (peginterferon beta-1a) for relapsing-remitting multiple sclerosis (RRMS) of one subcutaneous (SC) injection every 2 weeks is less frequent than other currently available injectable therapies [2–4].

The efficacy and safety of peginterferon beta-1a in patients with RRMS were demonstrated in the ADVANCE study, a 2-year, Phase 3, multicenter study with a 1-year placebo-controlled period. In Year 1, peginterferon beta-1a (125 mcg SC) administered every 2 or 4 weeks resulted in a significantly lower annualized relapse rate (ARR), risk of relapse, and 12-week confirmed disability progression than placebo, with a safety profile similar to established interferon beta-1a therapies [5]. After 96 weeks of treatment, ARR was further reduced in patients who continued on peginterferon beta-1a every 2 weeks, and was maintained in the every 4 weeks treatment group [6]. Magnetic resonance imaging (MRI) results reflected the clinical findings, showing that peginterferon beta-1a every 2 weeks significantly reduced the mean number of new or newly enlarging T2 hyperintense lesions, new T1 hypointense lesions, and gadolinium-enhancing (Gd+) lesions, and the mean volume of T2 hyperintense and T1 hypointense lesions, when compared with placebo and with the peginterferon beta-1a every 4 weeks treatment group at Week 24 and Week 48. In Year 2, there were further relative reductions in the number of new or newly enlarging T2 hyperintense lesions in both continuous peginterferon beta-1a groups compared with the delayed treatment group (patients who received placebo in Year 1, and were re-randomized to peginterferon beta-1a every 2 or 4 weeks in Year 2). The mean number of Gd + lesions at 2 years was significantly lower in the peginterferon beta-1a every 2 weeks group than in patients who crossed over from placebo to peginterferon beta-1a every 2 or 4 weeks at Week 48 (delayed treatment group) [6]. Overall, patients receiving continuous peginterferon beta-1a throughout the study displayed better efficacy than the delayed treatment group, and every 2 weeks dosing was more efficacious than every 4 weeks dosing. Peginterferon beta-1a was well tolerated across all treatment groups [6], and the safety profile was maintained over the 2 years. In total there were 9 deaths (7 had received at least 1 dose of study drug), which an independent data safety monitoring board concluded were not likely related to study drug and did not change the risk-benefit profile of peginterferon beta-1a [6].

As treatments have improved, achievement of minimal disease activity has become a realistic goal and ARR endpoints have become a less sensitive measure with which to evaluate new therapies in RRMS [7]. No evidence of disease activity (NEDA; also known as freedom from measured disease activity [FMDA]) is a new, more stringent clinical endpoint in MS incorporating both clinical and MRI aspects of disease activity, [7, 8] and has been evaluated in several clinical trials of disease-modifying therapies (natalizumab, cladribine, combiRx [interferon beta-1a and glatiramer acetate], fingolimod, peginterferon beta-1a), although there has been variation in the definition used [9–13]. Analysis of NEDA in Year 1 of ADVANCE showed that significantly more patients receiving peginterferon beta-1a every 2 weeks achieved NEDA compared with the peginterferon beta-1a every 4 weeks and placebo groups [13]. The objectives of the present analyses were to further explore MRI results, and determine the proportion of patients who showed NEDA over the full 2 years of the Phase 3 ADVANCE study.

## Methods

### Study design and participants

The study design has been described previously [5, 6]. Briefly, ADVANCE was a randomized, multicenter, double-blind, placebo-controlled Phase 3 cross-over study of peginterferon beta-1a in patients with RRMS [5]. Patients who met the following key eligibility criteria were recruited between June 2009 and November 2011: diagnosis of RRMS as defined by the McDonald criteria, [14] age 18 − 65 years, Expanded Disability Status Scale [15] (EDSS) score of 0 − 5, and at least two clinically documented relapses in the previous three years (at least one within the 12 months prior to randomization). Patients with progressive forms of MS, and those who had previously received interferon treatment for MS for longer than four weeks’ duration, or less than six months prior to baseline, were excluded. The protocol was approved by each site’s institutional review board and was conducted according to the International Conference on Harmonization Guidelines for Good Clinical Practice and the Declaration of Helsinki. Every patient provided written informed consent prior to study entry.

### Randomization and blinding

For the first year of the study, patients were randomly assigned (1:1:1) to receive SC injections of placebo, or peginterferon beta-1a 125 mcg every 2 weeks or every 4 weeks. To maintain dose blinding, all patients received an injection every 2 weeks; patients assigned to peginterferon beta-1a every 4 weeks received alternate injections of placebo and peginterferon beta-1a. During Year 2, all patients received dose-blinded peginterferon beta-1a, with patients initially randomized to active treatment continuing on the same dose regimen, and patients receiving placebo in Year 1 re-randomized to peginterferon beta-1a every 2 or 4 weeks at Week 48 [6].

All management, site personnel, investigators, and patients were blinded to treatment assignment. Each site used separate examining and treating neurologists, thereby maintaining blinding for all treatment groups.

### Study procedures, definitions and endpoints

Methods for the assessment of clinical and radiologic endpoints in the ADVANCE study after 1 and 2 years [5, 6] and details of additional Year 1 MRI analyses [13] have been published in detail previously. Here we describe methods relevant to the post-hoc analyses of MRI data collected throughout the 2-year study, and assessments of NEDA.

MRI scans obtained at screening and at Weeks 24, 48, and 96 were evaluated centrally in a blinded manner at NeuroRx Research, Montreal, Canada. MRI endpoints derived from MRI scans included: number and volume of T1-hypointense, T2-hyperintense and Gd + lesions; number of new active lesions (sum of Gd+ plus non-enhancing new or newly enlarging T2 hyperintense lesions); whole brain volume; and magnetization transfer ratio (MTR) in normal-appearing brain tissue (NABT) and Gd + lesions. A summary of pulse sequences acquired and the analysis methods are provided in Additional file 1 (Document S1: MRI acquisition parameters and analysis).

Overall-NEDA, MRI-NEDA, and clinical-NEDA were all evaluated for the periods 0–96 weeks and 48–96 weeks, and were defined as follows:

- Overall-NEDA: no evidence of clinical or MRI disease activity over the stated time period. This combines MRI-NEDA and Clinical-NEDA, both defined below.
- MRI-NEDA: no Gd + lesions at any scan after the beginning of the stated time period, and no new or newly-enlarging T2 hyperintense lesions at the end compared with the beginning of the period.
- Clinical-NEDA: no relapses^1^ and no onset of 12-week confirmed disability progression^2^ over the stated time period.

### Statistical analysis

The MRI analysis population comprised patients in the intent-to-treat (ITT) population who entered Year 2, consented to participate in MRI analysis and had any MRI data. Negative-binomial regression was used for analysis of new or newly-enlarging hyperintense lesions on T2-weighted images (adjusted for baseline number of T2 hyperintense lesions); multiple logit regression was used for the analysis of Gd + and new T1 hypointense lesions (adjusted for baseline number of respective lesions). The total number of new active lesions was determined based on Gd + and T2 lesion numbers without double counting. This was compared for each continuous peginterferon beta-1a group versus the delayed treatment group based on negative binomial regression, adjusted for baseline number of Gd + lesions. Changes from baseline in T2, T1 and Gd + lesion volumes, whole brain volume, and MTR of NABT were compared using a Wilcoxon rank-sum test. Post-hoc NEDA proportions were calculated directly, based on the definitions described above, using data from all eligible patients.

The primary analysis used last observation carried forward (LOCF) data (patients who did not have all measurements, but had no evidence of disease activity on any of the available measurements, were considered as NEDA). In a sensitivity analysis, patients who did not have all MRI measurements were excluded from the calculation of NEDA, even if they had no evidence of disease activity on available measurements. A logistic regression model was used to calculate odds ratios (ORs) and corresponding p-values for between-group comparisons. All data sets are available upon request.

## Results

### Patient disposition and baseline characteristics

Patient demographics and baseline characteristics in the overall study population were well balanced across the treatment groups; mean age was 36–37 years, 70–72% were women, and 81–82% were of white ethnic origin [5]. Key baseline disease characteristics relevant to the MRI and NEDA analyses are presented in Table 1. A total of 1332 patients completed Year 1 of the study and continued with active treatment in Year 2. The proportion of patients who completed Year 2 was similar across treatment groups: peginterferon beta-1a every 2 weeks, 391/438 (89%), peginterferon beta-1a every 4 weeks, 411/438 (94%), and delayed treatment 396/456 (87%) [6].

**Table 1:** Baseline disease characteristics (Calabresi et al. [5])

Characteristic	Placebo	Peginterferon beta-1a	
		Every 4 weeks	Every 2 weeks
All patients, n	500	500	512
Relapses in the 12 months prior to enrolment, mean (SD)	1.6 (0.67)	1.5 (0.62)	1.6 (0.67)
Baseline EDSS score, mean (SD)	2.44 (1.18)	2.48 (1.24)	2.47 (1.26)
T2 lesions at baseline			
Patients with available data, n	497	499	511
Mean number of lesions (SD)	50.6 (35.7)	51.4 (36.0)	48.7 (36.8)
Patients with no T2 lesions at baseline, n (%)	0	3 (<1)	4 (<1)
Gd + lesions at baseline			
Patients with available data, n	497	498	510
Mean number of lesions (SD)	1.6 (3.8)	1.8 (5.4)	1.2 (3.4)
Patients with no Gd + lesions at baseline, n (%)	296 (59.6)	297 (59.6)	334 (65.5)

*EDSS* expanded disability status scale, *Gd+* gadolinium-enhancing lesions, *SD* standard deviation

### MRI Outcomes

The numbers of new/newly enlarging T2 lesions and Gd + lesions during the 2 years of this study have been reported previously and are summarized in Additional file 2: Table S1 [6]. In brief, the mean number of new/newly enlarging T2 lesions from baseline to Week 96, and mean number of Gd + lesions at Week 96, were significantly lower with peginterferon beta-1a every 2 weeks compared with both delayed treatment and continuous peginterferon beta-1a every 4 weeks [6].

**Fig. 1:**
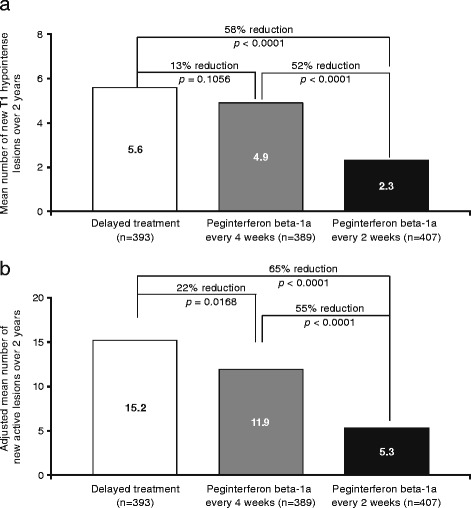
MRI lesions at Week 96: **a** new T1 hypointense lesions; **b** new-active lesions Gd+, gadolinium-enhancing lesions. MRI analysis population (ITT population dosed in Year 2 with at least 1 MRI result). **a** *P* values based on multiple logit regression, adjusted for baseline number of T1 lesions. **b** *P* values based on negative binomial regression, adjusted for baseline number of Gd + lesions

In the present analysis, we evaluated new T1-hypointense lesions over 2 years, and found that, again, significantly fewer T1 lesions formed during treatment with peginterferon beta-1a every 2 weeks compared with delayed treatment or peginterferon beta-1a every 4 weeks (58% and 52% reduction, respectively; both *p* < 0.0001; Fig. 1a). Patients in the peginterferon beta-1a every 2 weeks group also had, on average, significantly fewer new active lesions from baseline to Week 96 (65% and 55% reduction vs delayed treatment and peginterferon beta-1a every 4 weeks, respectively; both *p* < 0.0001; Fig. 1b). Additional file 3: Figure S1 shows two illustrative examples of Gd + and T2 lesions that developed into new T1-hypointense lesions over 2 years.

Total T2 hyperintense lesion volume decreased by a mean of 0.23 cm^3^ from baseline to Week 96 in the peginterferon beta-1a every 2 weeks group, while it increased in the other groups (mean increases of 0.62 cm^3^ in the delayed treatment group and 0.36 cm^3^ in the peginterferon beta-1a every 4 weeks; *p* < 0.0001 and *p* = 0.046, respectively, vs peginterferon beta-1a every 2 weeks; Table 2). A significantly smaller increase in T1 hypointense lesion volume was observed with continuous peginterferon beta-1a every 2 weeks compared with delayed treatment (0.48 cm^3^ and 0.87 cm^3^, respectively; *p* < 0.0001; Table 2). Gd + lesion volume decreased slightly in all groups, with no statistically significant difference between groups (Table 2).

**Table 2:** MRI lesion volumes at Week 96

Mean change from baseline (SD)	Delayed treatment	Peginterferon beta-1a	
		Every 4 weeks	Every 2 weeks
Patients with available data, n	391	389	406
T2 hyperintense lesion volume, cm^3^	0.617 (2.2341)	0.362 (2.6841)^†^	-0.231 (1.6103)^a^*
T1 hypointense lesion volume, cm^3^	0.869 (1.6907)	0.914 (2.4103)	0.478 (1.2417)*
Gd + lesion volume, cm^3^	-0.135 (0.5478)	-0.164 (0.8676)	-0.113 (0.4460)

*Gd+* gadolinium-enhancing lesions; *SD* standard deviation *p < 0.0001, ^†^ *p* = 0.046 versus delayed treatment by Wilcoxon rank-sum test ^a^ *n* = 407

**Fig. 2:**
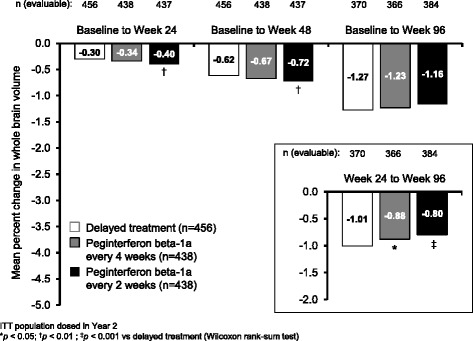
Percentage reduction in whole brain volume from baseline, and from Week 24 (*inset*). ITT population dosed in Year 2. *p < 0.05; ^†^ *p* < 0.01; ^‡^ *p* < 0.001 vs delayed treatment (Wilcoxon rank-sum test)

During the first year of the study, whole brain volume decreased from baseline to a greater extent with peginterferon beta-1a every 2 weeks than with delayed treatment (*p* < 0.01 at Weeks 24 and 48); however, the changes were small (<1%) and by Week 96, the reduction versus baseline was numerically smallest in the peginterferon beta-1a every 2 weeks group (Fig. 2). During the period from Week 24 to 96, reduction in whole brain volume was significantly smaller with both peginterferon beta-1a every 2 weeks and peginterferon beta-1a every 4 weeks compared with delayed treatment (Fig. 2 [inset]).

**Fig. 3:**
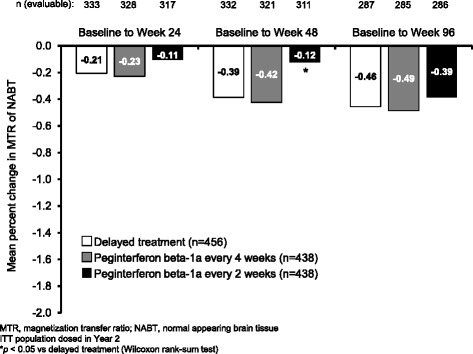
Percentage reduction in MTR of NABT. MTR, magnetization transfer ratio; NABT, normal appearing brain tissue. ITT population dosed in Year 2. *p < 0.05 vs delayed treatment (Wilcoxon rank-sum test)

MTR of NABT was reduced in all groups. At each time point, the reduction in NABT compared with baseline was smallest in the peginterferon beta-1a every 2 weeks group. At Week 48 (the end of placebo treatment for the delayed treatment group) MTR of NABT had decreased by a mean of 0.12% in the peginterferon beta-1a every 2 weeks group, compared with 0.39% in the delayed treatment group (*p* = 0.05; Fig. 3).

### Analyses of no evidence of disease activity (NEDA)

**Fig. 4:**
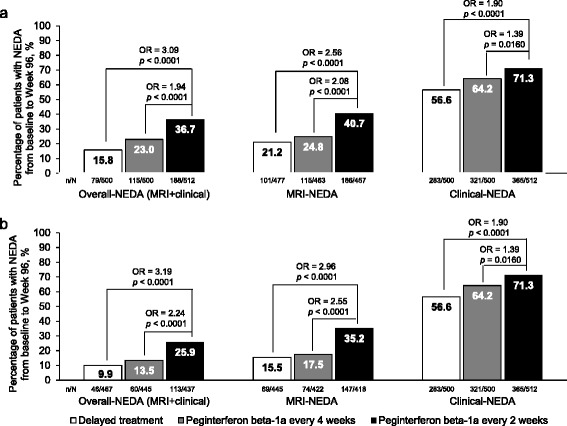
Proportions of patients with NEDA over 2 years (baseline to Week 96): **a** LOCF analysis; **b** observed data^a^. MRI, magnetic resonance imaging; NEDA, no evidence of disease activity; OR, odds ratio. ^a^Sensitivity analysis excluding patients with missing MRI data

Over the two years of the study, a significantly higher proportion of patients in the peginterferon beta-1a every 2 weeks group met overall-NEDA criteria compared with the delayed treatment group (36.7% vs 15.8%; OR 3.09; *p* < 0.0001). This was also significantly higher than the proportion in the peginterferon beta-1a every 4 weeks group meeting overall-NEDA criteria (23.0%; OR 1.94; *p* < 0.0001; Fig. 4a [LOCF analyses]). Both MRI and clinical components of NEDA were achieved by significantly higher proportions of patients in the peginterferon beta-1a every 2 weeks group compared with both delayed treatment and peginterferon beta-1a every 4 weeks (ORs for MRI-NEDA 2.56 and 2.08, respectively [both *p* < 0.0001]; ORs for clinical-NEDA 1.90 [*p* < 0.0001] and 1.39 [*p* = 0.016], respectively; Fig. 4a). Sensitivity analyses to exclude patients who did not have all MRI measurements for the calculation of NEDA were consistent with the primary (LOCF) NEDA analyses, with ORs the same or similar across all NEDA assessments (Fig. 4b).

**Fig. 5:**
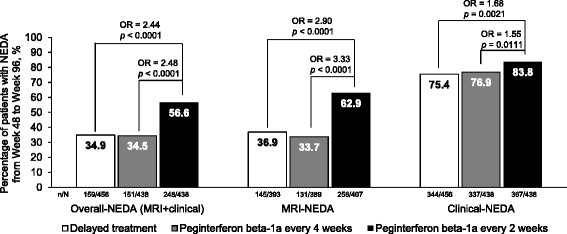
Proportions of patients with NEDA in Year 2 (Week 48–96). MRI, magnetic resonance imaging; NEDA, no evidence of disease activity; OR, odds ratio. LOCF analysis (includes patients who did not have all measurements, but had no evidence of disease activity on any of the available measurements). ITT population dosed in Year 2

The proportions of patients meeting criteria for overall-, MRI- and clinical-NEDA during Year 2 (Week 48 to Week 96) were higher in all groups than proportions achieving NEDA over the whole 2 years. Odds of achieving NEDA (overall and MRI and clinical components) remained significantly higher with continuous peginterferon beta-1a every 2 weeks compared with both delayed treatment (active treatment with peginterferon beta-1a every 2 or 4 weeks in Year 2) and continuous peginterferon beta-1a every 4 weeks (Fig. 5).

## Discussion

The outcomes from this analysis of MRI data and NEDA status are consistent with those data previously reported in the ADVANCE study, supporting the efficacy of peginterferon beta-1a dosed every 2 weeks. Continuous peginterferon beta-1a every 2 weeks consistently provided significant improvements in MRI lesion-based endpoints (including reduced numbers and/or volumes of new or newly-enlarging T2 lesions, new T1 lesions, Gd + lesions, and new active lesions) versus delayed treatment. Data from the first year of the ADVANCE study showed that improvements observed in many of these endpoints was statistically significant for peginterferon beta-1a every 2 weeks versus placebo at the first brain MRI scheduled at Week 24, and sustained through to Week 48 of the ADVANCE study [13]. Alongside recently published 2-year data from the same study, [6] which shows significantly lower mean numbers of new or newly-enlarging T2 and Gd + lesions when compared to delayed treatment or peginterferon beta-1a every 4 weeks, the analyses of T1 lesions and new active lesions presented here suggest that the effect of peginterferon beta-1a every 2 weeks on reducing mean numbers of lesions is further sustained throughout the 2-year study period.

We explored additional MRI measures besides lesions. Analysis of whole brain volume at 96 weeks did not reveal statistically significant treatment effects on brain atrophy. However, this analysis could have been confounded by the occurrence of pseudoatrophy, a phenomenon whereby brain volume decreases significantly during the first 6–12 months of anti-inflammatory treatment, possibly reflecting resolution of edema and inflammation present before treatment initiation [16]. Looking at change in brain volume from Week 24 to Week 96, (to eliminate the first 6 months of treatment in the continuous peginterferon beta-1a groups, when the impact of pseudoatrophy was likely to be greatest) the trend was reversed: there was a significantly smaller reduction in whole brain volume with both peginterferon beta-1a every 2 weeks and every 4 weeks, compared with delayed treatment. This suggests that peginterferon beta-1a could slow brain atrophy in the long term, although the 2-year overall treatment duration in this study was not sufficient to overcome the effects of pseudoatrophy in the first few months, and comparisons may be further confounded by onset of pseudoatrophy on initiation of treatment in Year 2 in the delayed treatment group. Furthermore, MTR measured in NABT over 96 weeks showed a trend for improved outcomes with continuous peginterferon every 2 weeks compared with delayed treatment. If reductions in brain volume over time were a result of true atrophy, it may be expected that this may also coincide with myelin loss (reflected by a decrease in MTR of NABT). Opposing trends for these measures during the first year of treatment with peginterferon beta-1a every 2 weeks (increased loss of brain volume coinciding with decreased reduction in MTR of NABT) support the suggestion that initial acceleration of loss of brain volume resulted from pseudoatrophy. A significant difference between peginterferon beta-1a every 2 weeks and delayed treatment in MTR of NABT change was apparent at Week 48, but was not sustained to Week 96; this may reflect a combination of attenuation of differences between the groups after peginterferon beta-1a treatment was commenced in the delayed treatment group and uncertainty in the point estimates given the small changes and variability in this measurement.

In addition to the MRI data, the present NEDA analyses support the efficacy of peginterferon beta-1a treatment every 2 weeks. Post-hoc analyses of efficacy data from ADVANCE showed that proportions of patients meeting criteria for overall-, MRI- and clinical-NEDA from baseline to Week 96 were significantly higher with peginterferon beta-1a every 2 weeks versus delayed treatment. MRI components of the NEDA composite appeared to be more sensitive to detecting treatment differences than clinical components; a high proportion of patients across all of the treatment groups met criteria for clinical-NEDA, supporting the value of including MRI components to support clinical-NEDA analyses. Nonetheless, NEDA criteria appear to be sufficiently robust to withstand missing MRI data, since the sensitivity analysis showed that inclusion of patients with missing MRI data in the LOCF analysis did not notably affect results. Currently, MRI-NEDA is defined as no Gd + lesions at Week 24, Week 48, or Week 96 and no new or newly-enlarging T2 hyperintense lesions compared with baseline over 96 weeks. Our analyses of MRI endpoints alongside post-hoc NEDA analyses support the use of this definition, since it reflects new active lesions detected on MRI scans. T1 lesion formation was also reduced during treatment with peginterferon beta-1a every 2 weeks, but any added value of incorporating this measure into the MRI-NEDA definition is not clear. We found limitations to potential incorporation of brain volume to the definition of NEDA: the small changes observed over periods of clinical interest, such as one or two years, and the noise in this measurement, as well as the complication of pseudoatrophy, make it unsuitable as a component of the MRI-NEDA and overall-NEDA composite endpoints.

Our results are consistent with our previous analysis of interim ADVANCE data, in which 39.8% of patients in the peginterferon beta-1a every 2 weeks group, and 17.8% in the placebo group, achieved overall NEDA in Year 1 (based on an LOCF analysis using the same NEDA definition as the current analysis) [13]. In the current analysis, overall NEDA rates over 2 years (baseline to Week 96) were 36.7% in the continuous peginterferon beta-1a group and 15.8% in the delayed treatment group. However, when we looked specifically at Year 2, these values increased to 56.6% and 34.9%, respectively. This supports the suggestion, based on our previous analysis, that it may be preferable to begin to assess NEDA status after a standard period of time (i.e., 6–12 months) following initiation of treatment, since MRI components may be affected by accrual of lesions early on before treatment efficacy becomes apparent [13].

The finding that more than half of patients on peginterferon beta-1a every 2 weeks achieved overall NEDA in Year 2 is encouraging, as recent longitudinal study in which NEDA status was monitored in a cohort of patients with MS over 7 years suggested that NEDA status at 2 years was a prognostic indicator for long term outcomes [17]. Future analyses of data from the ongoing ATTAIN study (an extension of ADVANCE) will reveal whether NEDA status is maintained for a high proportion of patients with longer-term peginterferon beta-1a treatment.

NEDA (sometimes referred to as FMDA or disease activity free [DAF]) has been assessed for many of the newer disease-modifying therapies becoming available for the treatment of MS. Rates are quite variable, with reported rates for overall-NEDA over 2 years ranging from 18% to 46% in trials of teriflunomide, dimethyl fumarate (DMF), fingolimod, natalizumab and cladribine [9, 10, 12, 18–20]. Rates should be compared with caution, as differences in study design, patient populations and MRI analysis techniques could contribute to variation, [8, 21, 22] although NEDA rates in placebo groups in these trials did not vary as widely as those in active treatment groups (7–16%). Considering differences in terms of ORs helps to account for differences in the sensitivity of the analyses, although ORs are not always provided in reporting of NEDA analyses. In a combined post-hoc analysis of data from the DEFINE and CONFIRM studies, the OR for achieving overall NEDA with DMF 240 mg twice daily versus placebo was 2.7 (23% vs 11%; *p* < 0.0001), [19] and in the CLARITY study ORs for cladribine 3.5 mg/kg and 5.25 mg/kg versus placebo were 4 · 28 and 4 · 62, respectively (44% and 46% vs 16%; both *p* < 0.0001) [10]. Peginterferon beta-1a administered continuously every 2 weeks for 2 years approximately tripled the likelihood of achieving overall-NEDA (OR 3.09) compared with delayed treatment.

Determining the relevance of this data to real-world experience will require further investigation, as patients are rarely completely lesion-free and have varying degrees of lesion activity. Interpretation is complicated by “noise” in the measurement of new lesions related to technical factors associated with the MRI acquisition and rater variability [23]. In part for these reasons, clinicians often look at a threshold for new lesions, which may be different between physicians, hospitals, and even different countries. A consensus on the use of MRI techniques and analysis may prove to be very beneficial to the study of MS, its pathophysiology, and potential treatments.

## Conclusion

Analyses of MRI outcomes and NEDA outcomes, along with efficacy for clinical endpoints previously reported, [5, 6, 13] show that the efficacy of peginterferon beta-1a is extended beyond the first year of the ADVANCE study, illustrating the benefits of peginterferon beta-1a on a number of different MRI outcomes as well as clinical-NEDA status. SC peginterferon every 2 weeks is the least frequently dosed interferon therapy for the treatment of multiple sclerosis, and the present analysis further supports the use of this therapy in patients with RRMS.

## Additional files

Additional file 1: Document S1. MRI acquisition parameters and analysis. (DOCX 27kb)
Additional file 2: Table S1. Summary of MRI endpoints over 2 years by original randomisation group [6]. (DOC 53kb)
Additional file 3: Gd + and T2 lesions that developed into T1 lesions: a) Gd + lesion that developed into a T1 lesion; b) new T2 lesion that developed into a T1 lesion. Figure A image 1 shows a Gd + lesion in the left prefrontal cortex at Week 48 that developed into a T1 lesion by Week 96 (image 2). Figure B image 2 shows a T2 lesion in the left prefrontal cortex at Week 48 that was not present at Week 24 (image 1). The lesion developed into a T1 lesion by Week 96 (image 3). Gd+, gadolinium-enhancing lesions. (JPG 2390 kb)

## Abbreviations

ARR: Annualized relapse rate
DAF: Disease activity free
DMF: Dimethyl fumarate
EDSS: Expanded Disability Status Scale
FMDA: Freedom from measured disease activity
Gd+: Gadolinium-enhancing
ITT: Intent-to-treat
LOCF: Last observation carried forward
MRI: Magnetic resonance imaging
MS: Multiple sclerosis
MTR: Magnetization transfer ratio
NABT: Normal appearing brain tissue
NEDA: No evidence of disease activity
OR: Odds ratio
PEG: Polyethylene glycol
RRMS: Relapsing-remitting multiple sclerosis
SC: Subcutaneous

## Footnotes

1 Relapses (confirmed by the independent neurologic evaluation committee) were defined as new or recurrent neurologic symptoms not associated with fever or infection, lasting for ≥24 h, and accompanied by new objective neurologic findings, and separated from the onset of other confirmed relapses by at least 30 days.

2 Disability progression was defined as an increase in the EDSS score of ≥1.0 point in patients with a baseline score of ≥1.0, or an increase of ≥1.5 points in patients with a baseline score of 0, confirmed after 12 or 24 weeks.
