# Supplementary material for: Peginterferon beta-1a improves MRI measures and increases the proportion of patients with no evidence of disease activity in relapsing-remitting multiple sclerosis: 2-year results from the ADVANCE randomized controlled trial
Source: BMC Neurol. 2017 Feb 10;17:29. doi: 10.1186/s12883-017-0799-0 (PMC5301356; doi:10.1186/s12883-017-0799-0)
Supplement: Additional file 1: Document S1. — MRI acquisition parameters and analysis. (DOCX 27kb) [file 12883_2017_799_MOESM1_ESM.docx]

**SUPPLEMENTARY MATERIALS**

**Document S1:** MRI acquisition parameters and analysis

**MRI pulse sequences:**

| **Hardware** | |
| --- | --- |
| Field strength | 1.5T  3T |
| Manufacturer | GE / Siemens / Philips / Toshiba |
| Model | Siemens: Symphony 1.5T, Sonata 1.5T, Avanto 1.5T, TrioTim 3T  Philips: Eclipse 1.5T, Achieva 3T  GE: Genesis 1.5T, Excite 1.5T, Excite HD 1.5T, HDx 1.5T, HDxt 1.5T, HDxt 3T  Toshiba: Atlas 1.5T |
| Coil type  (e.g. head, surface) | Quadrature or Multichannel (*) |
| Number of coil channels | If multichannel: 4 to 8 (*) |

| **Acquisition sequence** | |
| --- | --- |
| Type  (e.g. FLAIR, DIR, DTI, fMRI) | 2D Turbo Spin Echo  Proton Density weighted |
| Acquisition time | 2:30 to 5:00 |
| Orientation | Axial / Oblique |
| Alignment  (e.g. anterior commissure/poster commissure line) | AC-PC |
| Voxel size | 0.98 mm x 0.98 mm x 3mm (slice thickness) |
| TR | 2000ms to 3000ms (*) |
| TE | 7 to 15ms (*) |
| TI | - |
| Flip angle | 120° to 180° (*) |
| NEX | 1 |
| Field of view | AP: 250mm  RL: 187.5mm to 250mm (*) |
| Matrix size | 256 |

| **Acquisition sequence** | |
| --- | --- |
| Type  (e.g. FLAIR, DIR, DTI, fMRI) | 2D Turbo Spin Echo  T2 weighted |
| Acquisition time | 2:30 to 5:00 |
| Orientation | Axial / Oblique |
| Alignment  (e.g. anterior commissure/poster commissure line) | AC-PC |
| Voxel size | 0.98 mm x 0.98 mm x 3mm (slice thickness) |
| TR | 4264ms to 6330ms (*) |
| TE | 68ms to 91ms (*) |
| TI | - |
| Flip angle | 120° to 180° (*) |
| NEX | 1 |
| Field of view | AP: 250mm  RL: 187.5mm to 250mm (*) |
| Matrix size | 256 |

| **Acquisition sequence** | |
| --- | --- |
| Type  (e.g. FLAIR, DIR, DTI, fMRI) | 3D spoiled gradient echo  T1 weighted pre contrast |
| Acquisition time | 4:14 to 7:20 |
| Orientation | Axial / Oblique |
| Alignment  (e.g. anterior commissure/poster commissure line) | AC-PC |
| Voxel size | 0.98 mm x 0.98 mm x 3mm |
| TR | 28-30 ms (*) |
| TE | 4-11ms (*) |
| TI | - |
| Flip angle | 27° or 30° (*) |
| NEX | 1 |
| Field of view | AP: 250mm  RL: 187.5mm  SI: 180mm |
| Matrix size | 256 |

| **Acquisition sequence** | |
| --- | --- |
| Type  (e.g. FLAIR, DIR, DTI, fMRI) | 2D T2 Turbo Flair |
| Acquisition time | 7min to 9 min |
| Orientation | Axial / Oblique |
| Alignment  (e.g. anterior commissure/poster commissure line) | AC-PC |
| Voxel size | 0.98 mm x 0.98 mm x 3mm (slice thickness) |
| TR | 9000ms to 9820ms (*) |
| TE | 59ms to 97ms (*) |
| TI | 2000ms to 2500ms(*) |
| Flip angle | 120° to 180° (*) |
| NEX | 1 |
| Field of view | AP: 250mm  RL: 187.5mm to 250mm (*) |
| Matrix size | 256 |

| **Acquisition sequence** | | |
| --- | --- | --- |
| Type  (e.g. FLAIR, DIR, DTI, fMRI) | 3D spoiled gradient echo  T1 weighted post contrast | |
| Acquisition time | 4:14 to 7:20 | |
| Orientation | Axial / Oblique | |
| Alignment  (e.g. anterior commissure/poster commissure line) | AC-PC | |
| Voxel size | 0.98 mm x 0.98 mm x 3mm | |
| TR | 28-30 ms (*) | |
| TE | 4-11ms (*) | |
| TI | - | |
| Flip angle | 27° or 30° (*) | |
| NEX | 1 | |
| Field of view | AP: 250mm  RL: 187.5mm  SI: 180mm | |
| Matrix size | 256 | |
| Parallel imaging | ~~Yes~~ | No |
| If used, parallel imaging method:  (e.g. SENSE, GRAPPA) |  | |
| Cardiac gating | ~~Yes~~ | No |
| If used, cardiac gating method:  (e.g. PPU or ECG) |  | |
| Contrast enhancement | Yes | ~~No~~ |
| If used, provide name of contrast agent, dose and timing of scan post-contrast administration | 10’ post contrast  dose: according to the weight of the participant (0.1mmol/kg)  contrast agent could be:  prohance / magnevist / optimark / omniscan / multihance / Gadovist (*) | |
| Other parameters | Phase encoding direction: R/L  60 slabs | |

(*) Note that when a range or several values are provided, each individual scanner uses a fixed value.

**Image analysis methods:**

T2-weighted hyperintense lesions were segmented using locally developed software and manually corrected as necessary. To be counted lesions had to be ≥3 voxels in size. T1-weighted hypointense lesions were segmented on the post-contrast T1-weighted image using a threshold of 87% of the intensity of surrounding normal-appearing white matter, an intensity that is a little below that of cortical grey matter on the same image. Regions of acute T1 hypointensity associated with gadolinium enhancement were removed. New lesion counts were made relative to the prior visit and added over time intervals as appropriate. New active lesions were defined as the sum of Gd+ plus non-enhancing new or newly enlarging T2 hyperintense lesions. Magnetization transfer ratio (MTR) images were calculated from the percent difference between a T1w MRI and the same scan acquired with the addition of an off-resonance magnetization transfer (MT) preparation pulse. Normalized brain volume was determined at baseline, and percentage brain volume change calculated for each post-baseline MRI visit relative to baseline.
