# Supplementary material for: Peginterferon beta-1a improves MRI measures and increases the proportion of patients with no evidence of disease activity in relapsing-remitting multiple sclerosis: 2-year results from the ADVANCE randomized controlled trial
Source: BMC Neurol. 2017 Feb 10;17:29. doi: 10.1186/s12883-017-0799-0 (PMC5301356; doi:10.1186/s12883-017-0799-0)
Supplement: Additional file 2: Table S1. — Summary of MRI endpoints over 2 years by original randomisation group [6]. (DOC 53kb) [file 12883_2017_799_MOESM2_ESM.doc]

**Table S1:** Summary of MRI endpoints over 2 years by original randomization group [6]

| **Characteristic** | **Placebo** | **Peginterferon beta-1a** | |
| --- | --- | --- | --- |
|  |  | **Every 4 weeks** | **Every 2 weeks** |
| **All patients, n** | 500 | 500 | 512 |
| **New or newly enlarging T2 lesions at 2 years** |  |  |  |
| **Patients evaluated, n** | 393 | 389 | 407 |
| **Adjusted mean number of lesions** | 14.8 | 12.5 | 5.0 |
| **Lesion mean ratio (peginterferon beta-1a:delayed   treatment) (95% CI)^a^** |  | 0.84 (0.69. 1.03) | 0.33 (0.27, 0.41) |
| **p-value (peginterferon beta-1a vs. delayed treatment)^a^** |  | 0.0973 | <0.0001 |
| **Lesion mean ratio (peginterferon beta-1a every 2 weeks:   every 4 weeks) (95% CI)^a^** |  |  | 0.40 (0.32, 0.49) |
| **p-value (peginterferon beta-1a every 2 weeks vs.   every 4 weeks)^a^** |  |  | <0.0001 |
| **Gd+ lesions at baseline** |  |  |  |
| **Patients evaluated, n** | 393 | 389 | 407 |
| **Mean number of lesions (SE)** | 0.5 (0.08) | 0.7 (0.12) | 0.2 (0.06) |
| **p-value (peginterferon beta-1a vs. delayed treatment)^b^** |  | 0.2169 | 0.0002 |
| **Percent reduction (peginterferon beta-1a every 2 weeks   vs. every 4 weeks)^b^** |  |  | 71 |
| **p-value (peginterferon beta-1a every 2 weeks vs.   every 4 weeks)^b^** |  |  | <0.0001 |

CI, confidence interval; Gd+, gadolinium-enhancing lesions; SE, standard error

^a^Based on negative binomial regression, adjusted for baseline number of new or newly enlarging T2 lesions.

^b^Percent reduction based on group mean and p-value based on multiple logit regression, adjusted for baseline number of Gd+ lesions.
