# Supplementary figures and images for: Peginterferon beta-1a improves MRI measures and increases the proportion of patients with no evidence of disease activity in relapsing-remitting multiple sclerosis: 2-year results from the ADVANCE randomized controlled trial
Source: BMC Neurol. 2017 Feb 10;17:29. doi: 10.1186/s12883-017-0799-0 (PMC5301356; doi:10.1186/s12883-017-0799-0)

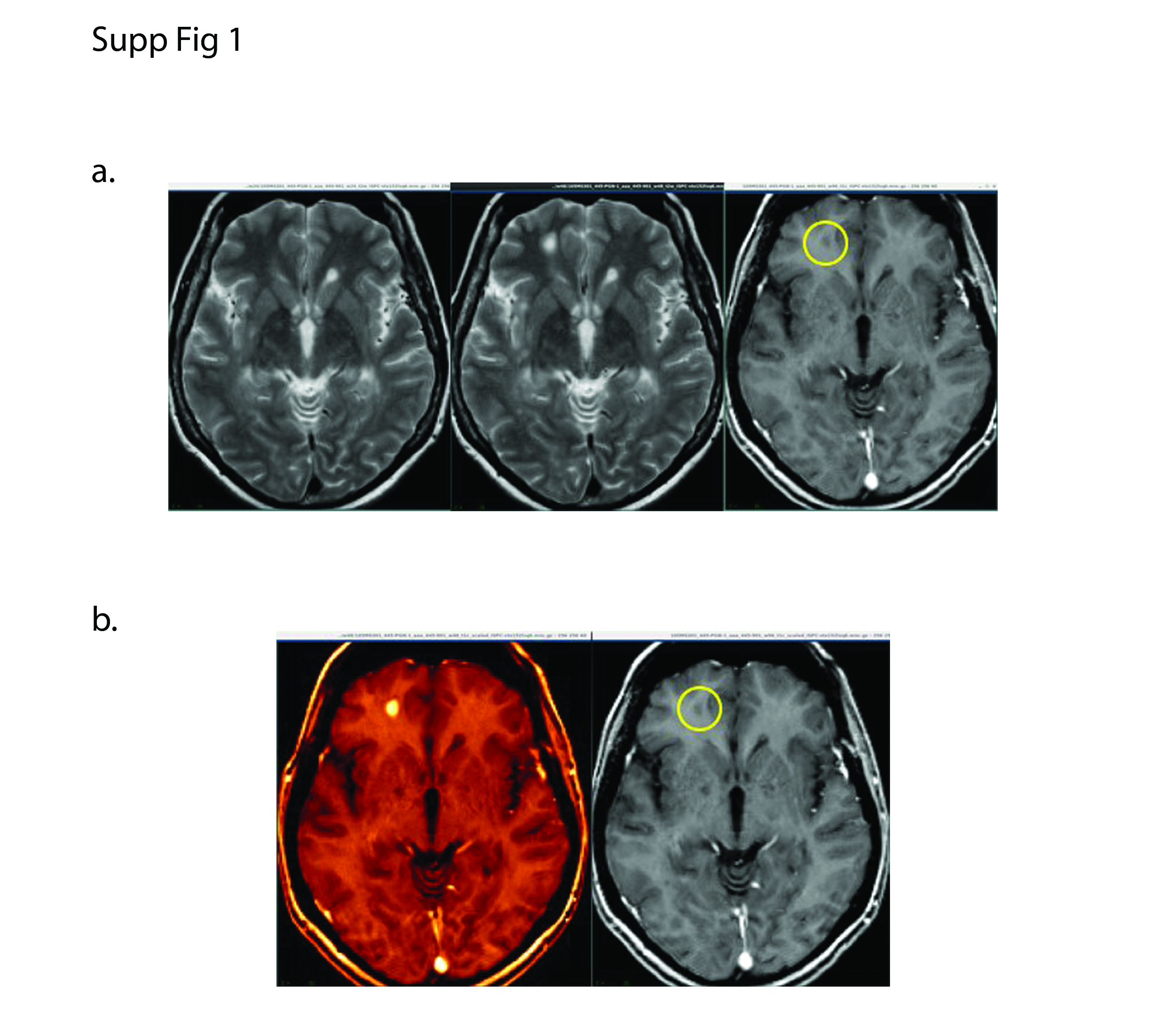

Supplement: Additional file 3: — Gd + and T2 lesions that developed into T1 lesions: a) Gd + lesion that developed into a T1 lesion; b) new T2 lesion that developed into a T1 lesion. Figure A image 1 shows a Gd + lesion in the left prefrontal cortex at Week 48 that developed into a T1 lesion by Week 96 (image 2). Figure B image 2 shows a T2 lesion in the left prefrontal cortex at Week 48 that was not present at Week 24 (image 1). The lesion developed into a T1 lesion by Week 96 (image 3). Gd+, gadolinium-enhancing lesions. (JPG 2390 kb) [file 12883_2017_799_MOESM3_ESM.jpg]
